# Do Native Parasitic Plants Cause More Damage to Exotic Invasive Hosts Than Native Non-Invasive Hosts? An Implication for Biocontrol

**DOI:** 10.1371/journal.pone.0034577

**Published:** 2012-04-06

**Authors:** Junmin Li, Zexin Jin, Wenjing Song

**Affiliations:** Institute of Ecology, Taizhou University, Linhai, Zhejiang, China; Centro de Investigación y de Estudios Avanzados del IPN, Mexico

## Abstract

Field studies have shown that native, parasitic plants grow vigorously on invasive plants and can cause more damage to invasive plants than native plants. However, no empirical test has been conducted and the mechanism is still unknown. We conducted a completely randomized greenhouse experiment using 3 congeneric pairs of exotic, invasive and native, non-invasive herbaceous plant species to quantify the damage caused by parasitic plants to hosts and its correlation with the hosts' growth rate and resource use efficiency. The biomass of the parasitic plants on exotic, invasive hosts was significantly higher than on congeneric native, non-invasive hosts. Parasites caused more damage to exotic, invasive hosts than to congeneric, native, non-invasive hosts. The damage caused by parasites to hosts was significantly positively correlated with the biomass of parasitic plants. The damage of parasites to hosts was significantly positively correlated with the relative growth rate and the resource use efficiency of its host plants. It may be the mechanism by which parasitic plants grow more vigorously on invasive hosts and cause more damage to exotic, invasive hosts than to native, non-invasive hosts. These results suggest a potential biological control effect of native, parasitic plants on invasive species by reducing the dominance of invasive species in the invaded community.

## Introduction

Invasive plants threaten human economic interests as well as the natural functioning of ecosystems and are thus a subject of active research within ecology [1]. Invasive plants are commonly exposed to complex environments in the recipient community, where many biotic and abiotic factors interact. The interaction between plants and their novel natural enemies is considered a central aspect of the mechanism that underlies the success of plant invasions and the control of invasive species [2].

Biological control (i.e., using natural enemies to control invasion success) has received much attention [3], [4]. The use of biological control agents is considered a “green” alternative for pest management, as a result of its effectiveness, low cost and relatively high environmental safety [5]. However, one of the serious ecological drawbacks of biological control is the introduction of more exotic species into new ranges. Alternatively, native enemies for exotic, invasive species may provide a viable control strategy [6]. If natural enemies cause more damage to exotic, invasive species than to native, non-invasive species, this strategy would be advantageous.

Parasitic plants commonly occur in many natural and semi-natural ecosystems in the world, where they play key roles in determining community structure and function and are considered keystone species [7]. Parasitic plants, especially holoparasitic plants, absorb nutrients and water from the host plant, which often reduces host performance, leading to a change in the competitive interaction between host and non-host plants and a cascade of effects on community structure and dynamics [8]. In 1965, parasitic *Cassytha filiformis* plants were used to control the invasive plant, *Bidens pilosa* [9]. Recently, *Cuscuta campestris*, *Cuscuta australis and Cassytha pubescens*, all parasitic species, have been considered as potential biological control agents for invasive plants [10]–[13]. Yu et al. conducted a field survey and found that parasitic *Cuscuta australis* exhibited more vigorous growth and a higher level of reproduction on invasive *Mikania micrantha* and *Wedelia trilobata* than on native plants [12]. In a field study, Prider et al. also found that the impact of parasitic *Cassytha pubescens* on the growth of invasive *Cytisus scoparius* plant was greater than the effect on native *Leptospermum myrsinoides* [13]. However, no empirical test has been conducted to quantify the damage caused by parasitic plants to invasive and native species and the possible mechanisms that cause invasive plants to be parasitized more readily than native plants.

Hosts with high growth rates would provide more susceptible tissue to parasites. Hosts with high resource use efficiency and high nutrient contents, such as legumes, are preferred hosts for parasitic plants [8], [14], but the impact of parasites on such hosts is also greater [15]. Thus, it is predicted that parasites may have a larger impact on invasive species than on non-invasive species. It is further predicted that the strength of the damage caused by parasites is positively correlated with the growth rate and resource use efficiency of its hosts. We studied 3 congeneric pairs of exotic, invasive and native, non-invasive herbaceous plant species grown with and without parasites in a greenhouse experiment in China to compare the biomass of parasites and the damage to exotic, invasive and native, non-invasive hosts. The relationships between the resistance, growth rate and resource use efficiency of the hosts were analyzed. We aimed to address the following three questions: (1) Can parasitic plants cause more damage to exotic, invasive plants than to native, non-invasive plants? (2) Is the level of damage related to the hosts' growth rate? (3) Is the level of damage related to the hosts' resource use efficiency?

## Materials and Methods

### Study system

We tested the effect of parasitic plants on their host plants. Three exotic, invasive plants and their corresponding congeneric native, non-invasive species were used. Plant species were selected based on their ability to germinate, their shared biological traits and similar size. *Bidens pilosa* and *B. bipinnata* are annual branching herbs belonging to the family Asteraceae. *B. pilosa* is native to tropical America and has spread widely in throughout China [16]. *B. bipinnata* is native to China and is widely distributed throughout the country [17]. Both plants commonly grow on cultivated land, hillsides and open waste areas.

*Solidago canadensis* and *S. decurrens* are perennial herbs belonging to the family Asteraceae. *S. canadensis* is native to North America and was introduced into China in 1935. It rapidly spread in South-East China, such as Zhejiang, Jiangsu, Jiangxi, Anhui, Hubei Province. It is often found in abandoned fields and along roadway. And *S. decurrens* is endemic to the southern China, such as Zhejiang, Jiangsu, Anhui and Guangdong Province [18].

*Ipomoea cairica and I. batatas* are perennial vines belonging to the family Convolvulaceae. *I. cairica* is native to East Africa [19] and has been introduced into Southern China [20]. *I. batatas* is one of the world's most important food crops and is widely planted in China. The majority of the world production of *I. batatas* occurs in China [21].

*Cuscuta chinensis* (Convolvulaceae) is an annual parasitic plant native to China that attacks more than 100 wild and cultivated species [22].

### Experimental design

We conducted a greenhouse experiment at Taizhou University (E 121°17′, N 28°87′) in Linhai City, Zhejiang Province, China. Seeds of native parasitic *C. chinensis* were purchased from the Chinese Herb Market (www.zgycsc.com). To cultivate the parasite, we used soybean as a temporary host plant for *C. chinensis*. Soybean seeds were purchased from Linhai City Vegetable & Seed Co., Ltd. (Linhai, China). We sowed uniform, healthy soybean seeds in farmland soil on May 20, 2008. When the soybean seedlings were approximately 10-cm tall, we sowed the seeds of *C. chinensis* in the soil around the soybean seedlings. *C. chinensis* successfully established on soybean after germination.

Pots that were 30 cm in diameter and 30-cm deep were filled with yellow, clay soil from a field in Linhai City (purchased from Mr. Ying, the owner of the field). Vegetation and litter were removed from the soil. The soil was mixed with sand in a 1∶1 ratio, with a final pH of 6.84±0.17, an organic matter content of 10.36±1.40 g/kg, an available nitrogen content of 27.90±8.08 mg/kg, an available phosphorus content of 31.88±9.34 mg/kg and an available potassium content of 42.20±3.35 mg/kg.

*Bidens pilosa* and *B. bipinnata* were geminated from seeds. Seeds of the invasive plant *B. Pilosa*, and the native plant *B. bipinnata*, were collected near the Sanfeng temple (E 121°16′, N 28°88′) on October 6, 2008. This is an open location owned by the government in Linhai City. The vegetation in this location is very common and no endangered or protected species are located here. On June 10, 2009, we sowed the seeds of *B. pilosa* and *B. Bipinnata* in trays containing sand for germination in a greenhouse. Two weeks later, 5-cm tall seedlings were transplanted into pots in a greenhouse.

*Solidago canadensis* and *S. decurrens* were propagated using direct transplantation of the seedlings collected from Taizhou City and Xianju County, respectively, in Zhejinag Province, China. The sites are located in an open, abandoned field and no specific permits were required for the described field studies. On June 25, 2009, intact, approximately 10-cm tall seedlings were collected with soil and moved immediately to the greenhouse. On the same day, the soil was carefully removed from the roots of the seedlings and transplanted into pots. The pots were set up in the greenhouse with shade to avoid excess transpiration. Sufficient water was irrigated in the pots every day, and three days after planting, shading was removed and the healthy seedlings were selected for future experiments.

*Ipomoea cairica* and *I. batatas* were propagated using cuttings. *I. cairica* plants were kindly provided by Dr. Zhao from Zhaoqing College in Guangdong Province. *I. batatas* plants were collected from Linhai City in Zhejiang Province (permitted by Mr. Ying, the owner of the plants). Both of them were successfully transplanted in the greenhouse before the experiment. On June 20, 2009, sharp pruning shears were used for cutting, after being sterilized with 70% ethanol. The upper parts of the healthy, disease-free plants were selected for cutting. Ten centimeter long cuttings were taken, and the remaining leaves were cut in half to reduce water loss. While maintaining the vertical orientation of the stems, we inserted the cuttings (one-third of their length) into pots containing soil into a greenhouse.

On July 20, 2009, 15-cm tall plants were selected for a completely randomized designed experiment. For factor parasitism, one case of parasitism and a control (without parasitism) were used. The stems of *C. chinensis* were cut into small pieces (15-cm long) and were twined onto the stems of both the invasive and the native hosts for infection. Six replicates were used for each treatment; a total of 72 pots were used in the experiment. The pots were randomly set up in the greenhouse and irrigated with tap water twice daily. The plants were fertilized with 1/4 Hoagland's nutrient solution [23] once per week. The temperature was maintained from 28°C to 30°C in the greenhouse.

### Measurements

At the beginning of the experiment (t1), six plants were harvested and separated into shoots and roots to obtain the original biomass (W1) and dried for at least 72 h at 70°C. On September 20, 2009 (t2), after two months of growth, the plants were harvested. *C. chinensis* was separated from its host and dried for at least 72 h at 70°C to determine the total parasite biomass. Harvested host plants were separated into shoots and roots. Leaves were scanned using an Epson Perfection 1670 Photo Scanner (Seiko Epson Corporation, Hino, Tokyo, Japan), and leaf area was measured with the WinFOLIA leaf area analysis system (Regent Instruments Inc., Quebec, Canada). Roots were washed and collected on a sieve with a 0.5 mm mesh screen, and an extra sieve (0.2 mm mesh) was placed at the outflow of the system to ensure that no fine root material was lost [24]. Debris and dead roots were manually removed from vital roots based on their colour and flexibility. To reduce root overlap and provide stability, roots were cut into 3 to 5 pieces, according to root size. Roots were immersed in water and scanned using the Epson Perfection 1670 Photo Scanner. The length and surface area of fine roots (diameter less than 2 mm) were measured and analysed with WinRHIZO root analysis system (Regent Instruments Inc., Quebec, Canada). After analysis, shoots and roots of host plants were dried for at least 72 h at 70°C to evaluate the shoot, root and total biomass (W2).

### Data analysis

In our study, the growth rate was reported as the relative growth rate (RGR) of the hosts, which was calculated according to the modified method by González-Santana [25], as RGR = (lnW2−lnW1)/(t2−t1), where W1 is the mean dry weight at time t1, and W2 is the plant dry weight at time t2.

Both leaves and fine roots are ephemeral and function primarily in resource acquisition [26]. The specific leaf area (SLA) is of great importance in regulating and controlling carbon assimilation and allocation [27] and is an indicator trait of the resource use strategies of plants [28]. Shen et al. found that SLA is tightly correlated with the resource capture and use efficiency of invasive plants [29]. In this study, SLA was used to indicate the leaf resource use efficiency and was calculated as leaf area/leaf dry mass [30]. Fine roots are essential for water and nutrient acquisition and are an important component of carbon flux in plants [31]. Fine roots may represent 33% of the global annual net primary productivity [32]. Fine root length and specific surface area are important indicators of nutrient cycling and resource capture [32]. In this study, specific fine root length (SFRL) and specific fine root surface area (SFRSA) were used to indicate the root resource use efficiency of hosts. SFRL was calculated as fine root length/fine root dry mass, and SFRSA was calculated as fine root surface area/fine root dry mass [33].

The strength of the parasitic plant damage to host plants was quantified by the deleterious effect (DE) that the parasites had on the host. The DE of parasites on a host was measured as the loss of fitness due to a given parasite infection. The DE was calculated as the difference in total biomass between parasitized plants and the mean total biomass of the control plants, standardized to the mean biomass of the control plants [34]; this value reflects the relative changes in host biomass caused by a parasite. A value of DE>0 indicates that parasitism facilitates the growth of the host, while a value of DE<0 indicates that parasitism inhibits the growth of the host. A value of DE = 0 indicates that parasitism had no effect on the growth rate of the host. The lower the value of DE, the stronger the negative effect of parasitism on the host.

To quantify the plastic responses of RGR, SLA, SFRL and SFRSA to parasites, the parasitism responses (PR) of these indices were also calculated as the difference in traits between parasitised plants and the mean of the control plants, standardised to the mean control plant levels, according to the following formula: PR = (parasitised-control)/control [34]. These values reflect the relative changes in traits of the hosts caused by parasites. A value of PR = 0 indicates no response of the plant to parasitism; a value of PR<0 indicates a negative response of the plant to parasitism; and a value of PR>0 indicates a positive response of the plant to parasitism.

A one-way analysis of variance (ANOVA) was used to analyze the effects of parasitism on plant species traits. A mixed-model, nested two-way ANOVA was used to analyze the effect of plant origin (invasive or native) and species (nested with origin) on the PR of host plants. Plant origin was used as a fixed factor, and species (nested with origin) was used as a random factor. A mixed-model nested three-way ANOVA was used to analyze the effects of parasitism (present or absent), plant origin and species (nested with origin) on host plant traits. Plant origin and parasitism were used as fixed factors, and species (nested with origin) was used as a random factor. Pearson correlation analysis was conducted to determine the relationship between the DE of parasites to their hosts and parasite biomass, as well as the growth rate and resource use efficiency of the hosts. SPSS (version 16.0) was used for all analyses, and *p*<0.05 was considered significant.

## Results

### The damage caused by parasites to invasive and native species

**Figure 1:**
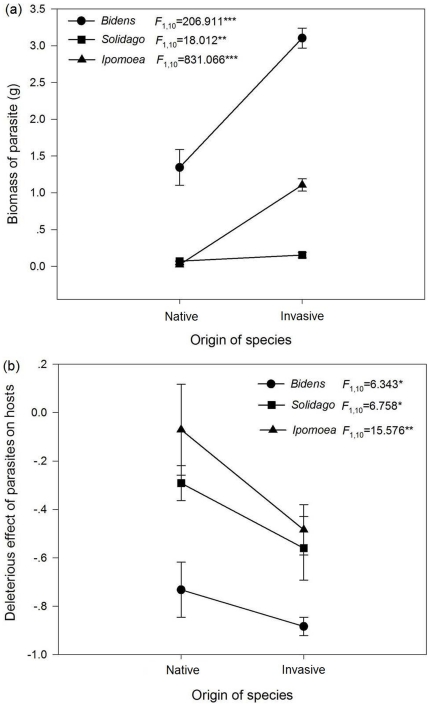
Means and standard errors of parasites biomass (a) and the deleterious effect of parasites (b) on exotic, invasive species and native, non-invasive species. *F*-values and significance levels of one-way ANOVA represent the effect of the origin of the species (invasive or native) on the parasites biomass and the deleterious effect of parasites on hosts (^***^ *p*<0.001;^**^ *p*<0.01; ^*^ *p*<0.05).

Parasites had significantly larger biomass (Fig. 1a) and caused significantly more damage (Fig. 1b) to exotic, invasive plants (*B. pilosa*, *S. canadensis* and *I. cairica*) than congeneric, native, non-invasive species (*B. bipinnata*, *S. decurrens* and *I. batatas*). Species (nested with origin) had a significant effect on parasite biomass (*F*_4,30_ = 471.427, *p*<0.001) and the DE of parasites to hosts (*F*_4,30_ = 9.134, *p*<0.001). The DE of parasites to hosts was significantly negatively correlated with the parasite biomass (*r* = −0.739, *p*<0.001), indicating that parasites growing more vigorously would have a greater impact on host plants.

### Correlation between parasite damage to hosts and host RGR

**Figure 2:**
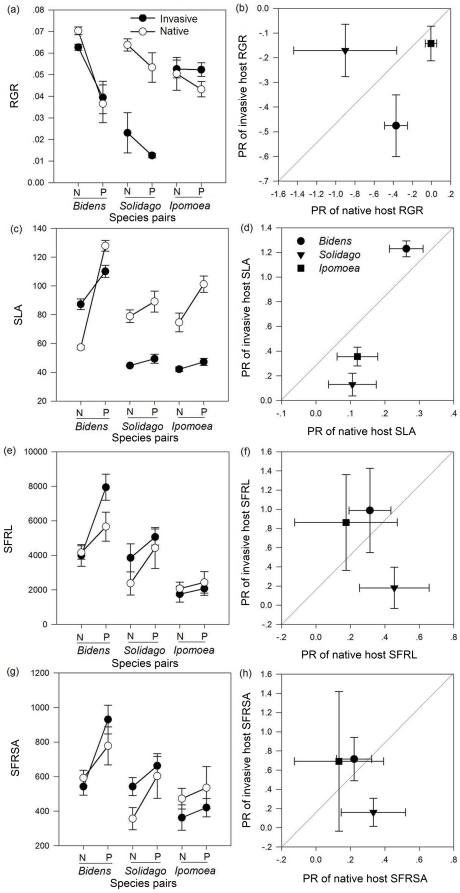
Growth rate and resource use efficiency of exotic, invasive and native, non-invasive plants and the corresponding parasitism responses. Means and standard errors of relative growth rate (RGR) (a), specific leaf area (SLA) (c), specific fine root length (SFRL) (e) and specific fine root surface area (SFRSA) (g) of invasive (filled circles) and native (open circles) species. Mean and standard errors of the parasitism response of RGR (b), SLA (d), SFRL (f) and SFRSA (h) of congeneric invasive and native species. A line indicates that there was no significant difference between the parasitism response of the traits of exotic, invasive species and native, non-invasive species. Points above or below the line show species-by-treatment combinations in which the trait of the exotic, invasive species was higher or lower than that of the native, non-invasive species.

Parasitism significantly decreased host RGR (Table 1), especially invasive *B. pilosa* (*F*_1,10_ = 72.324, *p*<0.001), native *B. bipinnata* (*F*_1,10_ = 56.543, *p*<0.001) and invasive *S. canadensis* (*F*_1,10_ = 11.928, *p*<0.001) (Fig. 2). Species (nested with origin) had a significant effect on host RGR (Table 1) and the PR of RGR (*F*_4,30_ = 15.825, *p*<0.001, Fig. 2), while plant origin had no significant effect on the PR of RGR.

**Table 1:** *F*-values and significance levels of three-way nested ANOVAs of the relative growth rate (RGR) and the resources availability of host plants with fixed factors parasitism (present or absent) and origin (invasive or native), and random factor species pairs (nested with origin).

Factors	Degree of freedom (df)	RGR	Specific leaf area (SLA)	Specific fine root length (SFRL)	Specific fine root surface area (SFRSA)
Parasitism (P)	1,64	**72.301** ***	**45.739** ***	**22.095** ***	**19.898** ***
Origin (O)	1,4	1.673	2.070	0.383	0.351
P×O	1,64	0.108	**5.316** *	0.765	**3.245** *
Species pairs (nested with origin)	4,64	**21.298** ***	**24.585** ***	**31.977** ***	**13.777** ***

Values in bold are significant at *p*<0.05; Significance indicated as follows:

* *p*<0.05,

** *p*<0.01,

*** *p*<0.001.

**Figure 3:**
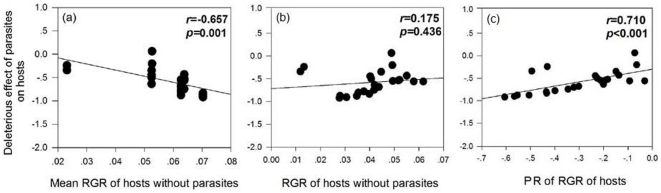
Correlation between the deleterious effect of parasites on hosts and the relative growth rate (RGR) of host with parasite (a) and without parasite (b), parasitism response of RGR of hosts (c). Pearson correlation coefficient (*r*) and *p*-values are given and values in bold are statistically significant at *p*<0.05.

The DE of parasites to hosts was significantly negatively correlated with the mean parasite-free host RGR and positively correlated with the PR of host RGR; however, no significant correlation was observed between parasite DE to hosts and the RGR of hosts with parasites (Fig. 3), indicating that parasites caused more damage to hosts with a higher RGR, and a larger RGR plastic response.

### Correlation between parasite damage to hosts and host resource use efficiency

Parasitism significantly increased the SLA, SFRL and SFRSA of hosts (Fig. 2 and Table 1). Species (nested with origin) had a significant effect on the SLA, SFRL and SFRSA of hosts, while the parasitism and origin interaction had a significant effect on the SLA and SFRSA of hosts (Table 1). Species (nested with origin) had a significant effect on the PR of the SLA, SFRL and SFRSA (*F*_4,30_ = 152.275, 11.708, and 13.820, *p*<0.001, Fig. 2).

**Figure 4:**
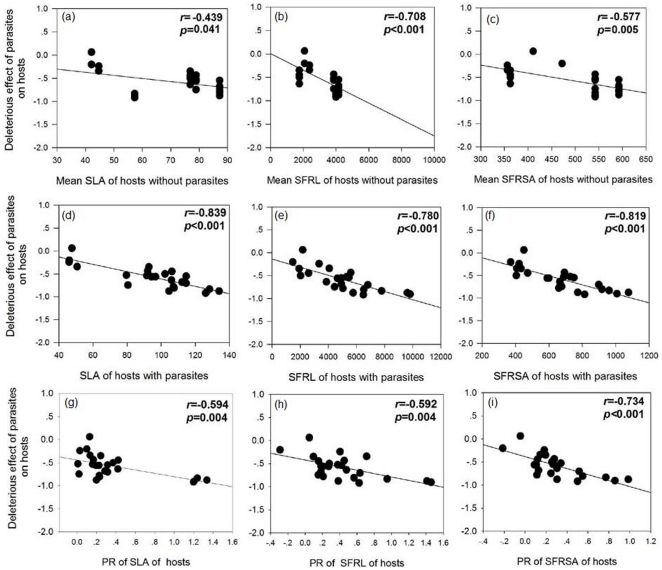
Correlation between the deleterious effect of parasites on hosts and the specific leaf area (SLA) (a), specific fine root length (SFRL) (b) and specific fine root surface area (SFRSA) (c) of hosts without parasites; SLA(d), SFRL(e) and SFRSA(f) of hosts with parasites; parasitism response of SLA(g), SFRL(h) and SFRSA(i) of hosts. Pearson correlation coefficient (*r*) and *p*-values are given and values in bold are statistically significant at *p*<0.05.

The DE of parasites to hosts was significantly negatively correlated with the SLA, SFRL and SFRSA of hosts with or without parasites. In addition, the DE of parasites to hosts was also negatively correlated with the PR of the SLA, SFRL and SFRSA of hosts (Fig. 4), indicating that parasites caused more damage to hosts with a higher resource use efficiency, and a larger plastic response of resource use efficiency to parasites.

## Discussion

The results revealed in this study confirmed our original hypothesis that the parasitic plant, *C. chinensis*, caused significantly more damage to exotic, invasive hosts than to congeneric native, non-invasive hosts. Our result is also supported by field studies reported by Yu et al., in which the parasitic plant, *C. australis*, exhibited more vigorous growth and higher reproduction on invasive plants (*Mikania micrantha* and *Wedelia trilobata*) than on native plants [12]. Prider et al. also showed that the impact of the parasitic plant *Cassytha pubescens*, on the growth of invasive *Cytisus scoparius* was greater than the impact on a co-occurring native plant, *Leptospermum myrsinoides* in the field [13].

Pennings and Callaway compared the interactions between parasitic plants and their hosts with the interactions between herbivores and plants and found that parasitic plants paralleled the host preferences of herbivores by reducing host biomass, altering host allocation patterns, modifying plant community structure and dynamics, and mediating interactions between host plants and other organisms [8]. A number of studies have shown that generalist herbivores performed better on invasive plants than native plants; in addition, they caused more damage to native plants than to invasive plants [35]–[37]. As a general parallel with herbivores, parasitic plants indeed grew vigorously and caused more damage to invasive species than to congeneric non-invasive species, as revealed in this pot experiment and other field studies [12], [13]. The damage caused by parasitic plants to hosts was significantly positively correlated with the parasitic plants' biomass, suggesting that invasive plants are more readily parasitized and more seriously damaged than native plants.

Our results directly demonstrated that the damage of the parasitic plant *C. chinensis*, to host plants was significantly positively correlated with the relative growth rate and the resource use efficiency of the hosts, indicating that the higher the RGR and resource use efficiency of the hosts, the more parasite damage there is to the hosts. It has been implied that the fact that exotic, invasive plants exhibit rapid growth, high levels of reproduction, and efficient resource capture and nutrient cycling contributes to their invasiveness [38]. Van Kleunen et al. conducted a meta-analysis and found that invasive species had higher leaf-area allocation and growth rates [39]. In the field survey, Yu et al. found that parasitic plant infection by *C. australis* was enhanced by the vigorous growth and high nutrient content of exotic, invasive hosts [12]. The vigorous growth and higher nutrient content of exotic, invasive hosts may underlie the mechanism by which parasitic plants grow more vigorously on invasive hosts and cause more damage to exotic, invasive hosts than to native, non-invasive hosts.

In addition, we also found that the parasite damage to hosts was significantly positively correlated with the plastic responses of SLA, SFRL, and SFRSA, whereas parasite damage was negatively correlated with the plastic responses of the RGR of parasitized hosts. In this study, parasitism significantly increased the SLA, SFRL and SFRSA of hosts, which can help hosts gain more resources to compensate for the biomass loss after damage, although the RGR of hosts was significantly inhibited by parasitism. The greater the host resource use efficiency, which was enhanced by parasitism, and the more the host growth was inhibited by parasitism, the greater the damage caused by parasitic plants to their hosts. Plants are highly plastic, and individuals within a species may vary by orders of magnitude in size, growth rate, allocation to different organs, reproduction, and chemical constituency [39]. Plants display morphological and physiological phenotypic plasticity in response to abiotic and biotic environments, including disturbance, herbivory, parasitism and mutualism [40]. It is often assumed that fast-growing species show more morphological plasticity than slow-growing species [41]. As a result, the inhibitory effect of parasitic plants on their hosts might be a vicious cycle: as parasitic plants absorb resources from their hosts, the hosts must reallocate additional biomass to leaves and roots to absorb more resources, which would then provide additional resources to the parasitic plants and lead to more severe host destruction. Press and Phoenix inferred that a particular host was preferred because of its abundance and its facilitation for the growth and development of parasitic communities [7]. This might be an explanation as to why the parasitic plant damage to hosts was positively correlated with the SLA, SFRL and SFRSA of hosts after damage. If parasitized hosts could provide more resources to parasitic plants and parasitism was preferred by the hosts, parasitic plants could cause more damage to their hosts.

In a field survey of the effect of a biological control experiment, artificial, introduced parasitic *C. campestris* could suppress the invasive plant *Mikania micrantha*, and contribute to native community recovery [11], [42]. The invasive plant *Alternanthera philoxeroides* was naturally infected and suppressed by the parasitic plant, *C. austrails*, which facilitated the recovery of its native community [43]. In addition, the parasitic plant *Cassytha pubescens* occurred at high densities on invasive plants and caused more damage to invasive species than to native species [13]. Our study showed that native, parasitic plants grow more vigorously on invasive hosts and cause more damage to exotic, invasive hosts than to native, non-invasive hosts; as a result, native parasitic plants have a potential biological control effect on invasive species by reducing the dominance of invasive species in the invaded community. For practical reasons, our pot experiment was conducted with fertilization to avoid differences in soil nutrient availability. Although resource nutrient availability might influence the magnitude of the host response to parasites and the damage caused by parasites to hosts, it does not limit us from extending our findings to the field, because invasive plants have high resource use efficiency, regardless of whether they are in high-resource environments or low-resource environments [44], [45]. As inferred by Prider et al., parasitic plants may not be abundant enough to resist initial invasion, but they may be an effective regulator of populations of invading species [13]. Parasitic plants could be an effective natural biocontrol agent for invasive species, and further research should focus on the ecological effect on all of the components in the invaded community, such as non-target, native species and underground microbial communities.
